# Evaluating the Educational Environment of a Nursing School by Using the DREEM Inventory

**DOI:** 10.5539/gjhs.v7n4p211

**Published:** 2015-01-14

**Authors:** Masoomeh Imanipour, Afsaneh Sadooghiasl, Shahrzad Ghiyasvandian, Hamid Haghani

**Affiliations:** 1 Nursing and Midwifery Care Research Center (NMCRC), Tehran University of Medical Sciences, Tehran, Iran; 2 School of Nursing and Midwifery, Tehran University of Medical Sciences, Tehran, Iran; 3 Statistics Department, School of Public Health, Iran University of Medical Sciences, Iran

**Keywords:** Educational Environment, DREEM, Dundee Ready Education Environment Measure

## Abstract

**Background:**

Educational Environment (EE) is considered as a key component of educational curriculum.

**Aim:**

The aim of this study was to evaluate the EE of Nursing and Midwifery School of Tehran University of Medical Sciences (TUMS) by using Dundee Ready Education Environment Measure (DREEM).

**Methods:**

This cross-sectional descriptive study was conducted in 2013. Totally, 500 nursing or midwifery students were recruited to the study by using the quota sampling method. Study data were collected by using a demographic questionnaire and the Persian version of DREEM questionnaire. The reliability of questionnaire was confirmed by Cronbach alpha which was 0.876 and 0.68–0.866 for the whole questionnaire and its domains, respectively. The data were analyzed by using SPSS v. 21.0.

**Results:**

Totally, 350 completely-filled questionnaires were included in the final analysis. Most of the participants were nursing students (79.7%), female (74.6%), single (86.0%), bachelor (86.9%), first-year (36.9%), with mean age of 22.5 years. The mean item score of the DREEM was 2.09±0.49 (104.39 from 200). Moreover, the mean item scores of the domains were as follows, perception of learning: 1.93±0.61; perception of teachers: 2.42±0.56; perception of educational atmosphere: 2.05±0.59; academic-self-perception: 2.06±0.65; and social-self-perception: 2.17±0.62. All domains were statistically significant except the perception of learning and educational atmosphere (p<0.001)

**Conclusion:**

Although the educational environment of the study setting was found to be positive, it requires improvements. Strategies such as adopting student-centered approaches, revising the educational curriculum, strengthening student-teacher relationship, being sensitive and responsive to students’ educational needs, providing constructive feedback to them, and creating a comfortable, friendly, and supportive atmosphere can improve the educational environment.

## 1. Introduction

Educational Environment (EE) is where teaching-learning process takes place. It includes physical, cognitive, cultural, psychosocial, emotional, educational, and motivational factors and provides a context for teachers and students’ teaching and learning activities. Accordingly, EE is considered as a key component and the spirit of educational curriculum (Tokuda et al., 2010).

EE is a central aspect of educational organizations because it has a substantial role in promoting students’ motivation, satisfaction, healthy competition, independence, self-confidence, learning, and critical thinking abilities (Soemantri, Herrera, & Requelme, 2010; Tokuda et al., 2010). Hammond et al. (2012) noted that EE—including both theoretical and clinical teaching-learning environments—has significant effects on students’ knowledge, attitude, practice, and behavior (Hammond, O’Rourke, Kelly, Bennett, & O’Flynn, 2012). Consequently, it is considered as an indicator of the quality of educational programs (Aghamolaei & Fazel, 2010). Al-Rukban et al. (2010) also found that students’ perceptions of and satisfaction with EE is directly related to their learning (Al-Rukban, Khalil, & Al-Zalabani, 2010).

Given the important role of students in teaching-learning process, their perceptions and evaluations of EE are considered as indicators of the effectiveness of educational curriculums. Each student has unique characteristics—such as previous educational experiences and preferred learning styles—and hence evaluates EE differently (Lorenzo & Lorenzo, 2013). According to Roff (1997), students usually take into account different factors such as their own learning, their social and academic selves, their teachers’ characteristics, and teaching-learning atmosphere when evaluating their EE. Accordingly, students who find their EE effective and appropriate usually achieve more satisfactory educational outcomes (Soltaniarabshahi, Kouhpayezadeh, & Sobuti, 2008).

Fulfilling the aims of education is among the main concerns of all medical education institutions. The primary mission of these institutions is to educate qualified workforce for meeting the health-related needs of society and therefore the graduates of these institutions are expected to have great skills and abilities for performing their professional roles. Accordingly, accreditation activities—including accreditation of the educational curriculum and EE—are usually performed for evaluating the effectiveness and the quality of educational endeavors of these institutions. The results of such accreditations provide valuable information which can be used for revising and improving the quality of educational curriculums.

EE is usually evaluated by using different methods and sources. Students’ views and perspectives on EE considerably contribute to identifying the characteristics of EE (Aghamolaei & Fazel, 2010). They are directly involved in EE and therefore have invaluable information about it. Moreover, they are the stakeholders of EE and have an important role in teaching-learning process. Accordingly, the revisions of educational curriculums should be made by using their views and perspectives (Lidice & Saglam, 2013).

Given the importance of educational evaluation and accreditation, reliable and appropriate instruments should be used for it. Currently, there are different instruments for this purpose. One of the most reliable instruments is the Dundee Ready Education Environment Measure (DREEM). DREEM was developed by Roff (1997). It evaluates EE by using students’ views. DREEM has five domains including students’ perceptions of learning (SPoL), students’ perceptions of teachers (SPoT), students’ perceptions of atmosphere (SPoA), students’ academic-self perceptions (SASP), and students’ social-self perceptions (SSSP). It can be used in different settings, contexts, and societies (Soltaniarabshahi et al., 2008). The aim of this study was to evaluate the EE of the Faculty of Nursing and Midwifery of Tehran University of Medical Sciences, Tehran, Iran, by using the DREEM.

## 2. Methods

This was a cross-sectional descriptive study conducted in 2013. The study setting was the faculty of nursing and midwifery of Tehran University of Medical Sciences, Tehran, Iran, with aim of evaluation of the faculty educational environment. This faculty was established in 1949. Currently, nursing and midwifery students are admitted to this faculty at bachelor, master and, PhD degrees. Moreover, international students are also studying nursing and midwifery in this faculty. The vision of the faculty is to achieve a superior educational status in the area. The study population consisted of all students affiliated to the study setting.

### 2.1 Study Population and Sampling

The study sample size was calculated by using a confidence interval of 0.95 and a precision of 3. Finally, to compensate the probable attritions, we recruited 500 students to the study. The sampling method of the study was quota sampling. Students’ level of education was considered as sampling quotas. Students who had filled the questionnaire incompletely were excluded from the study.

### 2.2 Research Tools

We collected the study data by using a demographic questionnaire and the DREEM. The demographic questionnaire consisted of items on students’ age, gender, marital status, residency status, field of study, educational level, year and semester of education, and the experience of other EE. The DREEM consists of 50 items in five domains including 1) students’ perceptions of learning (twelve items), 2) students’ perceptions of teachers (eleven items), 3) students’ perceptions of atmosphere (twelve items), 4) students’ academic-self perceptions (eight items), and 5) students’ social-self perceptions (seven items). Items were scored on a five-point likert-type scale ranging from completely disagree (0) to completely agree (4). Thus, the total score of the inventory would be 0–200. EE can be interpreted as very poor (0–50), has plenty of problems (51–100), more positive than negative (101–150), and excellent (151–200) based on the total score of DREEM (McAleer & Roff, 2000). However, to maintain a comparable unit of measurement, we divided the score of each domain by the number of items of that domain. Accordingly, the mean item scores of the DREEM and its domains ranged from 0 to 4. Item scores of higher than 3.5, between 2 and 3, and less than 2 are respectively interpreted as positive points, requires enhancement, and problematic. We translated the original English version of the DREEM into Persian and adjusted it to nursing and midwifery settings. We invited a panel of experts to assess and confirm the content validity of the inventory. Moreover, we evaluated the reliability of the DREEM by examining its internal consistency. The Cronbach alpha of the domains and of the whole inventory were 0.68–0.866 and 0.876, respectively.

### 2.3 Data Analysis

The data were analyzed by the Statistical Package for Social Sciences, SPSS v. 21.0. We used descriptive statistics measures such as mean and standard deviation for introducing the participants. Moreover, the repeated measures analysis of variance (RM ANOVA) and the Least Significant Difference (LSD) post-hoc tests were used for data analysis.

### 2.4 Ethical Considerations

The Nursing and Midwifery Care Research Center of Tehran University of Medical Sciences approved the study. We provided detailed information about the aim of the study for the participants. Informed consent was obtained from each participant.

## 3. Results

### 3.1 Demographic Status of Participants

Totally, 500 questionnaires were distributed to students. However, 150 incompletely filled questionnaires were excluded from the analysis. The response rate was 70%. Most of the participants were nursing student (79.7%), female (74.6%), single (86.0%), bachelor (86.9%), first-year (36.9%). The mean age of them was 22.5 years. Table 1 shows socio-demographic and educational characteristics of participants.

**Table 1:** Demographic and educational characteristics of participants

Items n=350	Number (n)	Percentage (%)
Gender		
Male	89	25.4
Female	281	74.6
Age (years)		
<20	144	41.1
20-30	177	50.6
>30	29	8.3
Marital status		
Single	301	86
Married	49	14
Residency		
Yes	127	36.2
No	223	63.8
Field of study		
Nursing	279	79.7
Midwifery	71	20.3
Level of education		
Bachelor	304	86.9
Master	25	7.1
PhD	21	6
Year of education		
First	36.9	129
Second	27.2	95
Third	26.8	94
Forth and more	9.1	32
The experience of other EE		
Yes	85	24.3
No	265	75.7

### 3.2 Scores of DREEM and Its Domains

The mean item score of the DREEM was 2.09±0.49 with total score 104.39 from 200. The mean item score of the domains were as follows, students’ perceptions of learning (SPoL): 1.93±0.61 (23.11 from 48); students’ perceptions of teachers (SPoT): 2.42±0.56 (24.25 from 44); students’ perceptions of atmosphere (SPoA): 2.05±0.59 (24.59 from 48); students’ academic-self-perceptions (SASP): 2.06±0.65 (16.48 from 32); and students’ social-self-perceptions (SSSP): 2.17±0.62 (15.17 from 28).

The results of RM ANOVA revealed that there was a significant difference between the domains of the DREEM. The results of LSD post-hoc test demonstrated that except SPoA and SPoL domains, the differences between all other domains were statistically significant (P value < 0.05; Table 2).

**Table 2:** The mean item scores of DREEM and its domains

Rank order	item	mean	±SD	median	range	Repeated Measure ANOVA test
	Total DREEM score	2.09	.45	2.06	0.94-3.44	Greenhouse-Geisser’s F=66.75 df=3.78 (p<0.001)
5	Students’ Perception of Learning	1.93	.61	1.91	0.33-3.42	
1	Students’ Perception of Teachers	2.42	.56	2.50	0.20-4.20	
3	Students’ Academic-Self Perception	2.06	.65	2.00	0.00-4.00	
4	Students’ Perception of Atmosphere	2.05	.59	2.00	0.33-3.75	
2	Students’ Social-Self Perception	2.17	.62	2.14	0.33-3.86	

## 4. Discussion

The results of the current study revealed a total score of DREEM was104.39 (out of 200) that means more positive than negative according to McAleer and Roff (2000). In a similar study carried out in the medical school of Hormozgan University, Iran, the overall score of DREEM was 99.6 (out of 200) (Aghamolaei & Fazel, 2010). Also, the results were similar to the studies conducted in Turkey (Demiroren, Palaoglu, Kemahli, Fozyurda, & Ayhan, 2008) and Saudi Arabia (Al-Rukban et al., 2010) that obtain an overall score of 117.63 and 119, respectively. However, the findings of other studies were different. For example the total score of DREEM was 139 in Dundee University (Al-hazimi et al., 2004). The score in a similar studies in Ireland (Hammond et al., 2012), and England (Varma, Tiyagi, & Gupta, 2005) was 127 and 139, respectively. Maybe dissimilarity of the educational systems has been caused these dissimilar findings.

Fidelma et al. (2006) noted that the DREEM score of schools that have modern educational systems would be higher than 120 (Fidelma, McAleer, & Roff, 2006). The other reason is that in this study the clinical educational setting of medical School was assess by DREEM.

In this study, five domains of EE were assessed by the DREEM questionnaire. The first domain was student perceptions of learning (SPoL). In this study, SPoL represented negative perceptions of students from teaching and learning. The lowest score in this area belonged to the ‘emphasis on long-term learning’ item. The score showed that long-term learning did not receive particular stress in this study setting. Currently, long-life learning is considered as an important outcome of education, therefore many strategies have been developed and implemented for promoting it. In recent decades, moving from teacher-centered to student-centered is one of these strategies. It is emphasizing on developing meta-cognition competencies such as critical thinking, problem solving, and independent learning abilities in students. These abilities are necessary for deal with nowadays changes and long life learning (Asoodeh, Asoodeh, & Zarepour, 2012; Chang, 2013).

Students’ perceptions of teachers was the next domain. The findings showed students believed their teachers are moving in the right direction. In this domain, except for the items “teachers provide constructive criticism here” and “teachers are good at providing feedback to students”, other items acquired a score of higher than 2. Giving constructive feedback to student is an essential task of teachers. It should be considered as one of the key characteristics of good teachers and also help to formative assessment of student’s performance (Plakht, Shiyovich, Nusbaum, & Raizer, 2013)

In students’ academic-self perceptions domain, the findings showed the presence of many negative aspects in EE. In this domain, items such as being well-prepared for the profession, being well-developed problem-solving skills, being able to memorize whatever required, and the last year working has been a good preparation for this year’s work—were below 2. The lowest score in this domain belonged to the “being well-developed problem-solving skills” item. One of the key objectives of student-centered educational programs is to develop students’ problem-solving skills. Problem-solving ability empowers students for effectively managing their problems later in their work life. Nursing students may encounter complex and difficult situations in their actual nursing practice that their management requires problem-solving ability. This ability helps nursing students to think critically, assess and, formulate their interventions logically (Choi et al., 2013).

According to this study, students’ perceptions of atmosphere showed there are many issues in EE that need to change. More active involvement of students in the teaching-learning process, as well as, creating more friendly and supportive climate were the most highlighted issues based on students’ believes.

Students’ social self-perceptions was the final domain that was investigated in this study. Based on the score, SSSP was “not too bad”. Items such as “having good friends in this course”, “having a good social life”, “being rarely bored in this course”, and “having good accommodation” were good with score higher than 2. Students believed there is no good support system for them when get stressed. They are too tired to enjoy the courses. According to literatures, providing strong support to students is one of the main responsibilities of educational systems, especially for vulnerable students. Some factors including entrance to an unfamiliar educational setting, having complex academic tasks, experiencing clinical and academic education, encounter with patients, concerns over future, can cause considerable stress among nursing and midwifery students. Therefore, Universities are responsible to create a supportive and caring EE because student would benefited from a compassionate and kind educational setting (Broadbent, Moxham, Sander, Sandra Walker, & Dwyer, 2014).

## 5. Conclusion

The study findings suggest that the students held higher expectations about their EE. Moreover, their perceptions from EE may considerably differ from their expectations. Accordingly, the EE of the study setting requires significant improvements. Adopting a student-centered approach to education can improve the EE and fulfill expectations of students. In summary, to have an effective education, students should be satisfied with EE. For this reason, recognizing the dimensions of EE is recommended.

**Limitations**

The study data were collected by using a self-report questionnaire which offers a subjective assessment of EE.
